# Ashwagandha (Withania somnifera (L.) Dunal) for promoting recovery in long covid: protocol for a randomised placebo-controlled clinical trial (APRIL Trial)

**DOI:** 10.1136/bmjopen-2024-094526

**Published:** 2025-04-25

**Authors:** Poppy Alice Carson Mallinson, Manisha Joshi, Mahesh Mathpathi, Alexander Perkins, Tim Clayton, Anoop SV Shah, Rohini Mathur, Nick Birk, Arandeep Dhillon, Judith Lieber, Sidra S Beg, Lily Hopkins, Archie Khan, Shereen Allaham, Vanessa TW Kam, Shailen Sutaria, Galib R, S Rajagopala, Amarjeet Bhamra, Geetha Krishnan G Pillai, Kamlesh Khunti, Tanuja Nesari, Sanjay Kinra

**Affiliations:** 1 Department of Non-Communicable Disease Epidemiology, London School of Hygiene & Tropical Medicine, London, UK; 2 Department of Global Health and Development, London School of Hygiene & Tropical Medicine, London, UK; 3 Centre for Global Chronic Conditions, Faculty of Epidemiology and Population Health, Department of Non-communicable Disease Epidemiology, London School of Hygiene & Tropical Medicine, London, UK; 4 Department of Medical Statistics, London School of Hygiene & Tropical Medicine, London, UK; 5 Centre for Primary Care and Public Health, Queen Mary University of London Wolfson Institute of Population Health, London, UK; 6 The University of Texas Health Science Center at Houston, Houston, Texas, USA; 7 Department of Infection Biology, London School of Hygiene & Tropical Medicine, London, UK; 8 Department of Epidemiology and Public Health, UCL, London, UK; 9 Aceso Global Health Consultants Ltd, London, UK; 10 Global Public Health Unit, Queen Mary University of London, London, UK; 11 All India Institute of Ayurveda, New Delhi, New Delhi, India; 12 All-Party Parliamentary Group on Indian Traditional Sciences, London, UK; 13 World Health Organization, Geneva, Switzerland; 14 Diabetes Research Centre, University of Leicester, Leicester, UK

**Keywords:** Safety, Primary Care, Post-Acute COVID-19 Syndrome, Randomized Controlled Trial, COMPLEMENTARY MEDICINE

## Abstract

**Background:**

Long covid describes a syndrome of persistent symptoms following COVID-19 and is responsible for substantial healthcare and economic burden. Currently, no effective treatments have been established. Ashwagandha (*Withania somnifera (L.) Dunal*) is a medicinal herb traditionally used in India for its immune-strengthening and anti-inflammatory properties. Withanolides, a family of steroid-derived molecules unique to Ashwagandha, have been shown to modulate inflammatory pathways in animal models, and several small randomised trials in humans support its effectiveness for reducing symptoms that are also associated with long covid. Therefore, this study aims to assess whether Ashwagandha is effective and safe for improving functional status and reducing symptom burden in adults living with long covid.

**Methods:**

A randomised double-blind placebo-controlled trial will be performed at participating general practice (GP) surgeries and long covid clinics across the UK. Individuals diagnosed with long covid will be screened for eligibility and then randomised 1:1 to take 1000 mg daily of Ashwagandha root extract tablets (standardised to <0.9% withanolides) or matching placebo tablets for 3 months (target, n = 2500). Monthly online surveys will be performed to collect patient-reported outcomes, and monthly safety monitoring, including liver function tests, will be conducted by clinical site teams. The primary outcome of the Post-COVID Functional Status Scale score at 3 months will be assessed by baseline-adjusted ordinal logistic regression, according to a pre-published statistical analysis plan. The secondary outcomes included validated quality of life and long covid symptom scales, work status and productivity and adverse events. The trial has been approved as a Clinical Trial of an Investigational Medicinal Produce by the Medicines and Healthcare Regulatory Authority and by the NHS Research Ethics Committee and Health Research Authority.

**Discussion:**

Treatments for long covid are urgently needed. This trial will robustly evaluate the safety and efficacy of a candidate treatment with a promising efficacy and safety profile. If found to be effective, the findings will likely influence treatment guidelines and improve health outcomes in those living with long covid.

**Trial registration number:**

This trial was pre-registered on 15/08/2022: ISRCTN12368131

**STRENGTHS AND LIMITATIONS OF THIS STUDY**

- This large-scale trial seeks to establish the safety and efficacy of a low-cost potential treatment for long covid, a priority disease area.
- Multicentre recruitment is being performed from clinical sites across the UK to reach a diverse patient population.
- Comprehensive clinical monitoring protocols will provide robust safety information.
- Trial outcomes are relevant patient-reported measures informed by patient involvement from an early stage.
- However, the study is limited to people with a registered diagnosis of long covid who can visit their general practice surgery or long covid clinic for monitoring visits, so they may not represent all long covid patients in the UK.

## Introduction

### Background and rationale {6a}

Estimates suggest that up to 45% of people with acute SARS-CoV-2 infection (COVID-19) globally may suffer some long-term symptoms,^1^ a syndrome commonly known as ‘long covid ’. A wide range of symptoms has been reported to occur in people with long covid, the most common being fatigue, muscle weakness, joint pain, respiratory problems (chronic cough, shortness of breath and chest tightness), cognitive dysfunction and poor mental health.^2 3^ The duration of symptoms of long covid can be highly variable, lasting sometimes for 1–3 months (‘ongoing symptomatic covid’), although in many cases persisting for substantially more than 3 months (‘post–COVID-19 syndrome’).^2^ Notably, over 70% of the 2 million people who reported living with long covid in the United Kingdom (UK) in March 2024 had been experiencing symptoms for at least 1 year,^4^ whereas a 2021 survey of long covid patients from 56 countries estimated that 91% of te respondents took more than 35 weeks to recover.^5^ This translates to a substantial health and economic burden globally. For example, care for individuals with long covid in England is estimated to cost almost four times more than before those individuals developed the condition,^6^ and approximately 80 000 people have left UK employment as a result of long covid.^7^

Currently, no effective treatments have been established for long covid. Identifying these is stated as a research priority in the UK’s National Institute for Health and Care Excellence (NICE) Guideline on long covid.^2^ While a few trials have indicated potential benefits of rehabilitation or cognitive behavioural therapy,^8^ the development of pharmaceutical targets for long covid has met limited success.^9^ The latest systematic review identified four randomised trials of drug interventions, which tested a disparate range of treatments (monoclonal antibodies, antidepressants, muramyl peptides and a calf blood extract), none of which provided clear evidence of benefit for long covid patients.^8^ This could in part be due to the broad and variable symptomology of long covid and the diverse target organs affected. Inflammation and immune dysregulation are proposed as central pathophysiological processes in long covid, although the mechanisms underlying these remain poorly elucidated.^10^

Ashwagandha (*Withania somnifera (L.) Dunal*) is a medicinal herb popular in the Indian Ayurvedic system of medicine proposed to have anti-inflammatory and immune-modulatory properties.^11 12^ It is traditionally used to promote energy and vitality, reduce stress and strengthen the immune system.^13^ Recently, several small randomised trials in humans have suggested its efficacy for improving muscle strength,^14 15^ exertional tolerance,^16^ sleep quality^17^ and cognitive function,^18^ reducing anxiety and stress^19^ and alleviating symptoms of fatigue in patients treated for chronic conditions.^20 21^ The pharmacological activity of Ashwagandha is primarily attributed to withanolides, a family of secondary plant metabolites derived from steroids, which are uniquely abundant in the *Withania* genus of plants.^22^ In vivo and in vitro studies have demonstrated the effects of withanolide compounds on inflammatory pathways including nuclear factor kappa B and Ak strain transforming, upstream of tumour necrosis factor-alpha (a key pro-inflammatory cytokine), demonstrating potential modes of action for Ashwagandha in immune and inflammatory conditions such as long covid.^11 12 23 24^

Based on extensive clinical use in India and over-the-counter use as an herbal supplement globally, Ashwagandha is believed to be well tolerated with minimal side effects.^25 26^ However, in the absence of rigorous pharmacovigilance or large-scale clinical studies, its efficacy and safety profile in clinical populations including long covid patients remains unestablished.

Thus, this study aims to generate robust evidence on the efficacy and safety of a standardised dose of Ashwagandha root extract to improve the functioning, quality of life and symptoms in adults with long covid.

This protocol is written in accordance with the Standard Protocol Items: Recommendations for Interventional Trials guideline for reporting of protocols of randomised controlled trials by following the structured study protocol template.

### Objectives {7}

The primary objective is to determine the effectiveness of a 1000 mg daily dose of Ashwagandha root extract tablets (<0.9% withanolides) for 3 months to improve functional status (measured by the Post-COVID-19 Functional Status Scale [PCFSS]) among people experiencing ongoing symptoms of COVID-19.

The secondary objectives include determining the effect of Ashwagandha tablets on the quality of life, reported fatigue, breathlessness, pain, sleep quality, mental health, cognitive function, work status, other long covid symptoms and adverse events (AEs).

### Trial design {8}

This is a randomised double-blind placebo-controlled superiority trial. The two trial arms (Ashwagandha and placebo) will be allocated on a 1:1 basis.

## Methods: participants, interventions and outcomes

### Study setting

This trial will be conducted through participating general practice (GP) surgeries and long covid clinics across the UK (collectively referred to as the trial sites). Participants will be recruited by their doctor (GP or long covid clinic doctor, hereafter referred to as the study doctor) randomised centrally and will receive the trial medication at their house by post. Monthly follow-up surveys will be conducted online, or by postal questionnaire, if preferred, and monthly clinical monitoring will be conducted by trial site clinical teams. The trial coordinating centre is the London School of Hygiene & Tropical Medicine (LSHTM) in London (UK).

### Eligibility criteria {10}

Eligibility will be assessed by a study investigator through a clinical screening assessment at participating trial sites.

Inclusion criteria:

- Adults (aged 18 years or older) with the capacity to provide informed consent
- Having been diagnosed with long covid as per the NICE Guidelines (NG188)^2^, that is, either one of ‘ongoing symptomatic COVID-19, with signs and symptoms of COVID-19 from 4 weeks up to 12 weeks’, or ‘post–COVID-19 syndrome, with signs and symptoms that develop during or after an infection consistent with COVID-19 that continue for more than 12 weeks and are not explained by an alternative diagnosis. It usually presents with clusters of symptoms, often overlapping, which can fluctuate and change over time and can affect any system in the body. Post–COVID-19 syndrome may be considered before 12 weeks while the possibility of an alternative underlying disease is being assessed. The diagnosis will be confirmed by the participant’s study doctor and/or medical records.
- Long covid reduced their ability to perform daily living activities compared with their ability before contracting COVID-19.
- Willingness and ability to complete the study protocols (take the trial medication regularly for 3 months, complete online telephone or postal surveys monthly and participate in clinical monitoring assessment monthly).
- Not taking any other herbal medicines, or willingness to stop taking any such medicines during the trial. Herbal medicine is defined as a plant or plant part, or mixture or extract of these, which is taken in medicinal form to improve health, prevent disease or treat illness.

Exclusion criteria:

- Self-diagnosed long covid in the absence of a clinical diagnosis as per the NICE guidelines.
- Any medical condition or suspected medical condition which, in the opinion of the investigator, may present an unreasonable risk to the study participant as a result of his/her participation in this clinical study (this may involve conduct of any clinical assessment deemed necessary by the study investigator to confirm that this criterion is met, such as (but not limited to) validated psychiatric scales, ECGs and laboratory tests for clinical chemistry, haematology, urinalysis, kidney function, etc).
- Previous clinical diagnosis of severe psychiatric disorders.
- Abnormal liver function test (LFT) results, as indicated by alanine aminotransferase or aspartate aminotransferase (AST) or total bilirubin (TBL) >2× the upper limit of the normal range (ULN), either measured as part of routine care within the past 3 months or conducted for the purposes of the clinical trial (if a recent test result is unavailable).
- Previous clinical diagnosis of chronic kidney disease or other medical condition associated with impaired kidney function.
- Previous clinical diagnosis of heart disease or other cardiac problems.
- Use of any investigational products within five elimination half-lives after the last dose or at screening.
- History of malignancy unless resolved by adequate treatment with no evidence of recurrence.
- Hypersensitivity to the active substance or to any of the excipients.
- Women breastfeeding or with a positive urine pregnancy test at screening.
- Women planning to become pregnant during the study period.
- Men and women of childbearing potential (WOCBP) unwilling to adhere to the relevant contraception requirements for the duration of the study (until at least 24 hours after the final dose of trial medication is taken). WOCBP are defined as all women who are fertile, following menarche and until becoming post-menopausal unless permanently sterile. Permanent sterilisation methods include hysterectomy, bilateral salpingectomy and bilateral oophorectomy. A postmenopausal state is defined as no menses for 12 months without an alternative medical cause. A high follicle-stimulating hormone (FSH) level in the postmenopausal range may be used to confirm a postmenopausal state in women not using hormonal contraception or hormonal replacement therapy. However, in the absence of 12 months of amenorrhea, confirmation with more than one FSH measurement is required. Acceptable contraception methods for WOCBP in this trial include combined hormonal contraception, progestogen-only hormonal contraception, intrauterine device, intrauterine hormone-releasing system, bilateral tubal occlusion, vasectomised partner, sexual abstinence or condom use. Sexual abstinence is defined as refraining from heterosexual intercourse during the entire period with risk associated with the study treatments. The reliability of sexual abstinence needs to be evaluated in relation to the duration of the clinical trial and the preferred and usual lifestyle of the participant. Male participants must use condoms. All participants must inform the investigator immediately if these contraception requirements are not met or if pregnancy is suspected.
- Participants taking benzodiazepines, anticonvulsants, barbiturates or any other central nervous system (CNS) depressants.

### Who will take the informed consent? {26A}

Informed consent to join the study will be requested from participants by a study investigator (or an appropriately trained and delegated member of their team) before the participant undergoes the clinical screening assessment. Informed consent will be sought only after the Participant Information Sheet has been shared at least 24 hours in advance, and a full explanation has been given by the investigator’s team with an opportunity to ask questions. Informed consent will then be obtained remotely or in-person, through an online form (with a box for the participant to add their free-hand signature) or paper form (which if remote may be posted to be completed by hand). All trial activities will be conducted according to the International Council on Harmonisation Good Clinical Practice (ICH GCP).

### Additional consent provisions for the collection and use of participant data and biological specimens {26B}

Any participant data or biological specimens collected as part of this trial will not be used in other studies. The consent form will include a request for permission for the study team to contact participants about participating in any future or follow-on studies should they be interested.

## Interventions

### Explanation for the choice of comparators {6b}

The investigational medicine product (IMP) being evaluated in this trial is an aqueous root extract of Ashwagandha in tablet form, taken orally at a daily dose of 1000 mg (4×250 mg tablets per day). The tablet formulation for this product is chosen because it is more amenable to blinding than other forms. The control arm will be an identical-looking placebo tablet based on microcrystalline cellulose to be taken as per the same regimen. A placebo tablet has been chosen to prevent participants’ awareness of treatment allocation from affecting their responses to the outcome questionnaires.

### Intervention description {11a}

The Ashwagandha and placebo tablets have been specially formulated for this trial by a reputed UK good manufacturing practice (GMP)-accredited pharmaceutical company based in India (Archimedis Healthcare Pvt. Ltd.), following extraction and validation methods described in the Ayurvedic Pharmacopoeia of India Part I Volume VIII.^27^ The trial batch of the active pharmaceutical ingredient was standardised to contain no more than 0.9% withanolides by content (validated by high-performance liquid chromatography). Both Ashwagandha and placebo are prepared as yellow-coloured circular tablets and packaged in white plastic bottles.

The medications will be sent by tracked courier to participants every month (every 30 days) for 3 months, from the trial coordinating centre. Participants will be requested to take two tablets each morning and two tablets each evening. Tablets should be swallowed whole, and if available, a glass of warm water can be used to wash them down (following Ayurvedic recommendations). Participants will be sent a medication diary each month in which they will be asked to keep track of their medication usage. If they miss a dose of medication, they will be asked not to catch up another time and to note the missed dose in the medication diary. Written instructions for how to take the medication will be provided to participants.

### Criteria for discontinuing or modifying allocated interventions {11B}

Participants will be required to immediately discontinue the trial treatment if any of the following criteria are met:

- Any serious AE (SAE, defined as a life-threatening illness, hospitalisation or onset of major disability) considered related to the study IMP.
- Any grade ≥3 (severe) or more severe AEs considered related to the study IMP.
- Pregnancy.
- Any medical condition that may jeopardise the participant’s safety if he or she continues study treatment.
- Use of prohibited medication as per the eligibility criteria.
- Liver toxicity:
  - Alanine transaminase (ALT) or AST >8× ULN.
  - ALT or AST >5× ULN for more than 2 weeks.
  - ALT or AST >3× ULN and (TBL >2× ULN or international normalised ratio >1.5).
  - ALT or AST >3× ULN with the appearance of fatigue, nausea, vomiting, right upper quadrant pain or tenderness, fever, rash and/or eosinophilia (>5%).

There are no planned intervention modifications in this trial.

### Strategies to improve adherence to interventions {11C}

If participants agree, regular text messages and/or email reminders will be sent (no more than weekly) to remind participants to take the tablets. They will be encouraged to use their trial medication diary to keep track of their medication usage, which may serve as a prompt for remembering to take the medication.

### Relevant concomitant care permitted or prohibited during the trial {11D}

Although there are no documented drug interactions for Ashwagandha, as a precaution based on its hypothesised mechanisms of action, the following medications are prohibited: benzodiazepines, anticonvulsants, barbiturates or any other CNS depressants. Clinical investigators have also been informed of a warning of potential interactions with the following medicines, which they will remain alert to during monthly clinical monitoring: immunosuppressants, thyroid hormones, antidiabetic drugs, antihypertensive drugs and aminoglycosides. Participants will be asked not to take any other herbal medicines during the trial. No other changes to participants’ lifestyle, medication or supplement use will be required as a result of joining this trial.

### Provisions for post-trial care {30}

Participants will not be offered any additional care beyond the stated duration of the intervention period (3 months). If any urgent health issues arise during the trial, participants are recommended to seek emergency care through their usual National Health Service (NHS) route and inform their study doctor as soon as possible. Participants may be entitled to compensation as a result of any harm arising from their participation in the trial, details of which will be given in the Participant Information Sheet.

If any participant becomes pregnant while taking the trial medication, the trial treatment will be immediately discontinued (as mentioned above). To understand if Ashwagandha has any effects on pregnancy, we will seek their consent to continue monitoring them until their due date to assess pregnancy outcomes. If the partner of a participant taking trial medication becomes pregnant, the participant may continue taking the medication; however, we will request permission to monitor the pregnant partner up until the due date to track the pregnancy’s outcome.

### Outcomes

The primary outcome is self-reported functional status measured using the PCFSS at 3 months.^28^ Functional status is a key patient-centred indicator that measures the extent to which a condition limits a person’s ability to carry out usual activities. The PCFS has gained widespread popularity as a tool for measuring self-assessed functional status in long covid in trials and observational studies and has been validated in long covid patients and translated into multiple languages.^29^,^31^ It was based on a similar, widely used functional scale for stroke patients.^32^ The PCFS asks participants to consider a flow chart of options that describe the extent of limitations to their daily activities over the past week. Based on the flow chart, they assign themselves to a single grade between 0 (no functional limitations) and 4 (severe functional limitations). The scale takes 1–2 min to complete. The outcome will be analysed at the end of the study (3 months) using ordinal logistic regression, with adjustment for the participant’s baseline (pre-intervention) value.

The secondary outcomes are as follows: i) quality of life by Patient-Reported Outcomes Measurement Information System (PROMIS) 29+2 summary score v2.1,^33^ ii) self-reported fatigue, pain, sleep, anxiety, depression and social, physical and cognitive function by PROMIS 29+2 individual dimensions, iii) self-reported breathlessness by an adapted version of the modified Modified Medical Research Council Dyspnoea Scale,^34 35^ iv) health utility by the EuroQoL 5-Dimension 5-Level utility score,^36^ v) work status and productivity and vi) other self-reported symptoms. Outcome domains were selected to cover the most relevant key indicators for patients and policy makers as well as the most common symptoms of long covid. Outcome questionnaire instruments were chosen following a thorough review of the literature and other ongoing studies on the basis of validation/wide use, short completion time (to reduce participant burden) and relevance for this study population (ideally have been used successfully in long covid patients).

All primary and secondary outcomes are patient-reported and will be collected via online questionnaires shared with participants on a monthly basis (or by postal questionnaires if the participant prefers).

AEs (harm) will also be assessed between trial arms based on the results of monthly clinical monitoring by the participant’s study doctor (see Adverse Event Reporting section for more details).

### Participant timeline {13}

**Figure 1:**
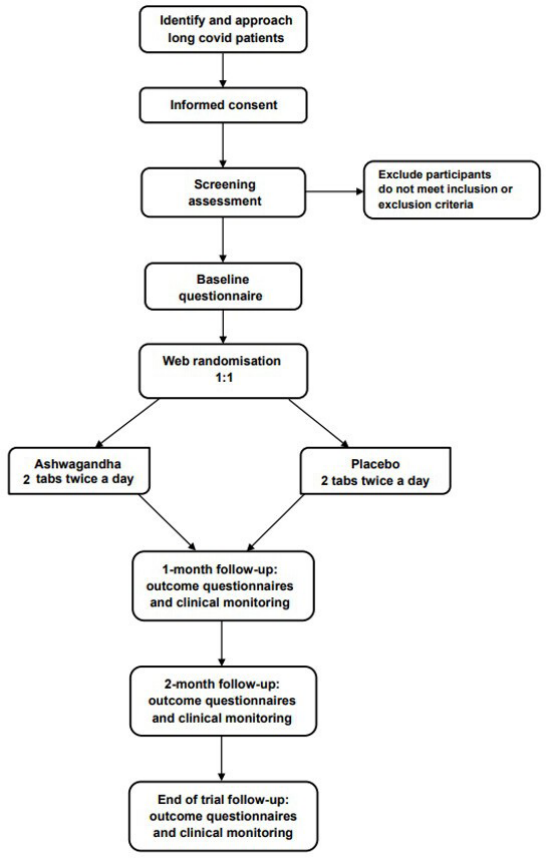
Flow chart of trial recruitment and follow-up in the APRIL Trial.

**Figure 2:**
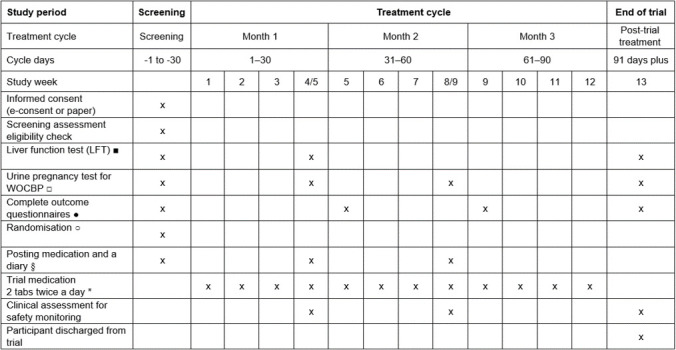
Schedule of trial activities in the APRIL Trial. ■ Liver function test (LFT) – if the blood test has been already conducted as part of standard of care within the past 3 months then it can be used and there is no need to repeat further test unless clinically indicated. Follow-up blood tests to be done within 7 days of target date. □ Urine pregnancy test for women of child-bearing potential (WOCBP) – urine pregnancy test will be done at screening, after completing month 1 and month 2 trial treatment and at the end of the treatment to confirm nil pregnancy. If a female subject becomes pregnant during the treatment period, she should immediately notify the investigator and trial medication should be permanently discontinued. ● Monthly outcome questionnaires to be completed within 2 weeks of completion of month 1, month 2 and month 3 treatment. ○ Randomisation must be completed within 2 weeks of confirmation and signing of participant eligibility. § To post month 2 and month 3 trial medication along with the trial medication diary a week prior the start of cycle. * To start treatment within 7 days of randomisation.

Each participant will take part in the trial for 3–4 months (depending on the time taken in the pre-treatment stage). A flowchart of trial recruitment and follow-up is given in figure 1, and the detailed schedule of trial activities is given in figure 2. In summary, participants will provide consent and undergo eligibility screening (including clinical assessment, medical history, baseline LFT and urine pregnancy test for WOCBP) with their study doctor. Once confirmed as eligible, they will be sent an online questionnaire to complete (baseline questionnaire). They will be electronically randomised into the trial once the questionnaire is completed. They will be sent their trial medication by post as soon as possible after randomisation and contacted to confirm receipt and the day of medication initiation (day 0). After 30 days of taking the trial medication, they will have received their second pack of trial medication by post and an online questionnaire to complete and will meet with their study doctor’s team for clinical monitoring (including month 1 LFT) (±3 day window). After 60 days of taking the trial medication, they will have received their third pack of trial medication and a link to their online questionnaire to complete and will have a clinical monitoring assessment with their study doctor’s team (±3 day window). After 91 days, participants will complete their final online questionnaire and clinical monitoring assessment with their study doctor (including month 3 LFT) (+7 day window) and be discharged from the trial (unless any clinical concerns have arisen, which might indicate further clinical monitoring). WOCBP will additionally undergo urine pregnancy tests at each monthly monitoring assessment to confirm that they are not pregnant while on trial medication (for which month 1 and 2 monitoring assessments may be done at home and reported to the study doctor’s team).

### Sample size {14}

Published data from an online survey of long covid patients’ grade distribution on the PCFS (4 [severe] to 0 [none]) improved from 3.4%, 49.4%, 35.1%, 7.1% and 5.0% at 3 months to 2.1%, 43.9%, 35.1%, 10.5% and 8.4% at 6 months, respectively.^29^ If Ashwagandha can improve symptoms to a similar extent at 3 months, this translates to an overall OR of 0.71 on the ordinal logistic regression analysis (with an OR <1 indicating a shift to a lower grade of symptoms). To improve symptoms with a more modest OR of 0.75, a sample size of 2500 gives in excess of 90% power (or 80% power to detect an even more conservative OR of 0.80). An OR of 0.75 would result in the percentages in each grade (grades 4 to 0) being 2.6%, 43.1%, 38.8%, 9.0% and 6.6% in the Ashwagandha arm at 3 months. The primary analysis will include an adjustment for the baseline value of the primary outcome scale, which is expected to increase the power further. These calculations are designed to be robust to limited anticipated loss to follow-up (given the relatively short-term and non-onerous nature of the trial).

### Recruitment {15}

Participants will be recruited by a clinical investigator based at the trial’s participating sites. Trial site teams will search their local patient databases for potentially eligible individuals and contact them by letter or in person to invite them to join the trial.

New study sites will be recruited by the trial coordinating centre (LSHTM) through letters of invitation or personal communications and networks, as appropriate.

### Assignment of interventions: allocation

#### Sequence generation {16A}

Participants will be randomised online in a 1:1 ratio to the intervention or control arms. A randomisation list will be generated by the independent trial statistician, which will include a list of randomisation IDs and their corresponding trial allocation, using randomly permuted blocks with random block sizes to ensure balance throughout the recruitment period.

#### Concealment mechanism {16B}

The randomisation list will be used by an independent labelling partner at a GMP facility in the UK (IMP Pharmaceuticals Ltd) to apply blinded labels to all the bottles. The blinded bottles will be sent to the trial coordinating centre (in random order) to be dispatched to participants according to their assigned randomisation ID. None of the staff involved in running the study will have access to the unblinded randomisation lists. Neither participants nor any staff involved in running the trial will be made aware of the participant’s randomisation status.

#### Implementation {16C}

Described above.

## Assignment of interventions: blinding

### Who will be blinded? {17a}

Participants; study staff conducting the recruitment, handling participant communications and collecting outcome information; and study investigators and analysts will all be blinded to the participant’s assignment status. Only the independent unblinded staff member (responsible for generating the randomisation lists and the unblinded statistical reports to the data monitoring committee [DMC]), and the independent labelling partner (not otherwise involved in the study) will be aware of the assignment of interventions. The DMC will be unblinded for SAE monitoring and can also request unblinded interim analyses of outcomes (to be prepared by the independent unblinded statistician).

### Procedure for unblinding if needed {17b}

In general, there should be no requirement for unblinding of the allocated treatment in this trial. If a participant wishes to withdraw, or a new contraindication to Ashwagandha arises, a withdrawal process will be followed (but unblinding will not occur). In exceptional circumstances, the study investigator responsible for a particular participant may need to unblind that participant (eg, in a clinical emergency where clinical management requires knowledge of which treatment the participant has received). In the case of such eventualities, all study investigators will have access to unrestricted and immediate access to break the treatment code via a password-protected online system that allows them access to only the requested participant’s treatment code. The responsibility to break the treatment code in emergency situations resides solely with the investigator. An electronic logging system will be in place to alert the trial team to all occurrences of unblinding of an investigator so that this can be logged and followed up appropriately.

### Data collection and management

#### Plans for assessment and collection of outcomes {18A}

Follow-up for primary and secondary outcomes will be through a brief monthly online or postal survey. All questionnaire instruments are previously validated (full versions available in the appendix). Questionnaires will be sent monthly for 3 months (ie, end of months 1, 2 and 3). To be valid, completed questionnaires should be received within 2 weeks of the target completion date (but ideally as close to the target date as possible). Automated email or text reminders will be sent as per participant preference. In case participants are unable or unwilling to complete surveys online, paper questionnaires in pre-paid return envelopes will be posted each month along with the supplement/placebo packages.

The study doctor’s team will assess all participants at least monthly and conduct symptom-guided assessment as described in NICE Long Covid guidelines. Assessments will take place at the end of 30 ± 3 days (first interim assessment), 60 ± 3 days (second interim assessment) and 91 (to 98) days (final end of trial assessment) from treatment initiation. At each assessment, they will actively elicit the participant’s history related to a) any worsening of their long covid, b) exacerbation of any other pre-existing conditions and c) any side effects that might be related to the drug (including AE/SAE assessment if applicable). To guide assessment of c), we will provide the clinical teams with a list of possible side effects that have been observed previously for this trial product (regardless of whether causally attributed). Monthly assessments could be conducted remotely or in-person, based on requirements (eg, if blood tests are required). Following the initial assessment, further in-person follow-up, including any indicated tests and investigations, will be sought if needed based on the participant’s symptom assessment and as guided by relevant local and national guidelines. In addition to these monthly clinical assessments, LFTs (AST, ALT and bilirubin) will be conducted at 30 (ie, along with first interim clinical monitoring assessment) and 91 (ie, at the end of trial assessment) days. The test at 30 days is intended to check for any early acute liver toxicity associated with the trial medication; if there is any abnormality noted, participants will be assessed on-site. The test at 91 days must be performed at least 24 hours after the last dose of the trial medication and is intended to assess for any longer-term effects associated with the cumulative build-up of the product in the system. All WOCBP will have to complete a urine pregnancy test monthly during the trial. These tests will be done after completing month 1 and 2 trial treatments and after completing month 3 treatment at the end-of-study assessment to confirm that no pregnancies occurred during the trial treatment phase. The tests will be posted directly to the women and may be done at home or at the participant’s trial site. All clinical and laboratory measures will be made as per the trial site’s standard NHS clinical protocols to ensure that the assessment of these safety outcomes complies with local clinical standards.

### Plans to promote participant retention and complete follow-up {18B}

Automated email or text reminders will be sent as per participant preference. If participants fail to respond for 2 months in a row, follow-up calls will be made to them (no more than three times) to ascertain their participation status in the trial (including vital status). If participants are withdrawn from the treatment (eg, at their own request or as recommended by their study investigator), we will give them the option to continue completing the online follow-up questionnaires if they wish (referred to as ‘partial withdrawal’).

### Data management {19}

Completed informed consent forms will be retained by clinical trial sites as per their standard procedures. Data from screening, registration and follow-up case report forms will be stored on secure servers within the trial coordinating centre’s secure data centre. These data systems will be access-controlled with access only provided to necessary study staff such as those entering and monitoring the data, and trial staff based at study sites will only be able to view information pertaining to participants from their site. Any paper-based case report forms will be stored in locked cabinets at the trial coordinating centre. Where data entry is required (ie, for any sections completed by post or telephone), entry into the same database (via Redcap forms) will be overseen and checked by the trial manager. The database will be developed in a way that flags up unusual data values, allowing participants to be contacted by the trial team via phone if there are large amounts of unusual/missing data. Once the trial data entry is completed and all data queries have been satisfactorily resolved, the database will be locked down by the trial manager by revoking site staffs’ access and downloading the final version of the database as an un-editable file stored on the sponsor’s secure servers. Data and all appropriate documentation will be stored for a minimum of 10 years after the completion of the study, including the follow-up period.

### Confidentiality {27}

All personal information about potential and enrolled participants will be stored securely at the trial sites or trial coordinating centre in accordance with general data protection regulations and following stringent confidentiality guidelines. It will only be accessible to necessary study personnel on a need-to-know basis (eg, for conducting participant follow-up and posting trial medications), following relevant consents from participants. Only de-identified data required for conducting trial analyses will be shared with the study analysts. These data extracts will be de-identified because no participant identifiers (eg, name and date of birth) will be included, and participants’ trial IDs will be replaced with new random IDs. They will be extracted from the main locked study database by the trial manager and shared with the statistical analyst via shared folders within the sponsor’s secure servers. Data handling will be in accordance with standards set out in the UK NHS Data Security and Protection Toolkit.

### Plans for collection, laboratory evaluation and storage of biological specimens for genetic or molecular analysis in this trial/future use {33}

Blood sample collection for LFT (which must include at a minimum ALT, AST and TBR) will be organised by the recruiting investigator’s team and will be conducted as per their clinic’s usual NHS testing arrangements. The trial site research nurse or phlebotomist will draw approximately 5 mL (one teaspoon) of venous blood from the participant’s arm in a gold top blood collection bottle. This blood sample will then be sent to the site’s local NHS testing laboratory for analysis. After the blood has been analysed, the NHS laboratory will destroy the blood sample following NHS standard policy for human tissue destruction, in accordance with the Human Tissue Act.

Urine pregnancy testing will be conducted either by the investigator’s team when the participant visits the trial site or by the participant at home. If the urine pregnancy test is conducted at the trial site, then the test result will be analysed and documented by the research nurse or delegated individual performing the urine pregnancy test, and the urine sample will be disposed of by trial site staff following local human tissue disposal protocol. If the test is being conducted at home, the participant will be sent urine pregnancy kits to their home address and provided instructions on conducting the test. They will be asked to report their test results to their study doctor or research nurse for documentation.

Biological samples collected as part of clinical safety monitoring for this trial will not be retained for future use.

## Statistical methods

### Statistical methods for primary and secondary outcomes {20A}

A detailed statistical analysis plan will be developed and signed off before any unblinded analyses are undertaken. The primary analysis (of end-line PCFSS grade) will be on an intention-to-treat basis using ordinal logistic regression for ordinal scale data with patient-reported outcomes,^37 38^ adjusted for baseline levels of the outcome. Secondary outcomes will be analysed by ordinal (for ordered categorical variables), logistic (for binary variables) or linear (for continuous variables) regression, also adjusted for baseline values where applicable. Further secondary analyses will include multivariate models that adjust for baseline covariates: age, sex, presence of cardiometabolic comorbidity and other baseline factors that exhibit imbalance between trial arms at baseline (should there be any).

### Interim analyses {21B}

Unblinded interim analysis of AEs will be undertaken by the independent DMC regularly during the trial to monitor the safety of the intervention according to the DMC Charter. Based on unblinded results, the DMC will make a recommendation to the trial steering committee (TSC), who will be responsible for making a decision based on the DMC’s advice (subject to approval by the trial sponsor and/or regulatory body where applicable).

### Methods for additional analyses (eg, subgroup analyses) {20B}

Secondary analyses will involve limited pre-specified subgroup analyses for sex, ethnicity, symptom severity at enrolment and symptom duration at enrolment. We will also examine participant trajectories of primary and secondary outcomes over the treatment period using data from all 3 months of outcome surveys.

### Methods in analysis to handle protocol non-adherence and any statistical methods to handle missing data {20C}

For all primary and secondary analyses, treatment allocation will be assumed to be ‘as randomised’ (intention-to-treat). An exploratory per-protocol analysis may also be conducted for comparison purposes. Multiple imputations will be used to handle missing data.

### Plans to give access to the full protocol, participant-level data and statistical code {31C}

The protocol, questionnaires and statistical code for the analyses will be made available via the trial website. De-identified extracts of participant-level data will be made available following an embargo period (reserved for analysis by the study team) and subject to participant consent and satisfactory anonymisation criteria being achieved.

### Oversight and monitoring

#### Composition of the coordinating centre and trial steering committee {5D}

The trial management group (TMG) will be responsible for day-to-day management and decision making for the trial; this will be comprised of the study chief investigator (CI), trial manager and relevant study co-investigators (Co-Is) as required. They will meet monthly or more often as required. The TSC will be responsible for the overall oversight of the trial and key decisions including approval of changes to the protocol; this will be comprised of an independent chair, two other independent members, one Co-I, the CI and two patient representatives. They will meet approximately every 3 months during the trial.

### Composition of the data monitoring committee, its role and reporting structure {21A}

An independent DMC comprised of three clinical trial experts (external to the study) has been established prior to trial initiation. This committee will be primarily responsible for monitoring the safety of participants during the trial. They will conduct regular unblinded analyses of AEs (approximately every 3 months), detailed in the DMC Charter, which will be developed prior to the start of recruitment. They will be able to request additional interim analyses with appropriate justification in the interest of participant safety. They will pass any recommendations arising from interim analyses onto the TSC.

### Adverse event reporting and harms {22}

Participants will be provided with contact details for their recruiting site’s clinical team to report any potential adverse reactions/events as they occur. Site clinical staff will also prompt participants to report previously unreported AEs during the monthly clinical assessments. Potential AEs and their symptom, location and duration reported during monthly assessments will be entered into an AE assessment form, which will include an assessment for seriousness and causality by a study investigator and any recommendation follow-up/monitoring (if applicable). A report summarising all non-SAEs will be provided to the DMC every 3 months. All SAEs will be reported to the sponsor within 24 hours of the trial team becoming aware of them, regardless of their assessed causality.^1 2^

### Frequency and plans for auditing trial conduct {23}

The study may be subject to auditing by LSHTM (under their remit as sponsor) and other regulatory bodies, such as the Medicines and Healthcare Regulatory Agency (MHRA), to ensure adherence to the trial protocol and ICH GCP.

### Plans for communicating important protocol amendments to relevant parties (eg, trial participants and ethical committees) {25}

All protocol amendments will be approved by the^1 4^ to the MHRA, HRA, LSHTM ethics committee and NHS Research Ethics Committee for review. Participants will be informed (via email, post or phone) only if the amendment is relevant to them (eg, duration or dosage of supplement or frequency of contact), and additional consent will be sought as necessary.

### Dissemination plans {31A}

Primary study results will be disseminated via a peer-reviewed publication in an open-access high-impact journal as soon as possible after trial databases are locked. Following this, reports to long covid patient groups, press releases, conference presentations and other public statements may be made. Additional publications arising from the trial (secondary analyses, etc) will also be published in peer-reviewed journals.

### Patient and public involvement

People with long covid provided input into the overall trial design and recruitment methods (at the project conception stage), choice of outcome measures and design of the outcome questionnaires (at protocol development stage) and trial management and oversight by holding voting positions in the TSC (during trial implementation).

## Discussion

The APRIL trial will robustly evaluate whether 1000 mg of Ashwagandha root extract, taken daily for 3 months, can help improve functioning and alleviate symptoms of people diagnosed with long covid. Given the scale of the long covid crisis and the lack of effective treatment options,^39^ this trial will generate vital evidence around a potential therapy that could be readily and rapidly available to long covid patients should it be demonstrated to be effective and safe.

The trial design and its ongoing recruitment have been made possible through strong support and input from representatives of long covid patients and healthcare providers. For example, we conducted patient interviews to inform the outcome measures and pilot test trial questionnaires, engaged with long covid civil society organisations and primary care providers to inform our protocol and recruitment strategies and have two long covid patient representatives on our TSC to ensure ongoing patient oversight of the trial. These interactions have consistently highlighted the ongoing burden of long covid for patients and the healthcare system, the frustration of patients and providers on the limited treatment options available and an eagerness to contribute to medical research, which they worry is being increasingly sidelined since the acute COVID-19 pandemic has subsided. We also noted that many long covid patients in the UK (and elsewhere) are already experimenting with over-the-counter Ashwagandha supplementations, highlighting the urgent need for clinical safety data on Ashwagandha for this patient population so that regulators and clinicians can better advise patients about the use of this product.

The key strengths of the trial are as follows: a large trial with multicentre recruitment from clinical sites across the UK to reach a diverse patient population, comprehensive clinical monitoring protocols to provide robust safety information, rigorous randomisation and blinding procedures and use of an extensively researched battery of patient-reported outcome measures as the primary outcomes, informed by patient involvement from an early stage. A key limitation of the trial is that it only includes people with a registered diagnosis of long covid who are attending a GP surgery or long covid clinic. The comparison of GP diagnosis figures with Office of National Statistics survey data reveals that numerous individuals with long covid in the UK may not be diagnosed, limiting the generalisability of our findings to these patients.^40^ In addition, some long covid patients with severe activity limitations may not be able to visit the clinical sites for LFTs and any other required in-person monitoring activities; this was unavoidable as LFT was a regulatory requirement for this trial, at least until more robust safety data becomes available. Our initial plan was to conduct the trial remotely to avoid this issue, but this was modified based on a request from the MHRA to collect comprehensive safety data at clinical sites initially, before revisiting the decision to conduct remote recruitment and follow-up. As such, our findings may not generalise to patients with the most severe symptoms of long covid.

In conclusion, we present a large, double-blind randomised placebo-controlled clinical trial of Ashwagandha (*Withania Somnifera (L.) Dunal*) to help people recover from long covid. If found to be effective, the trial results could inform treatment guidelines for people suffering from long covid globally.

## Trial status

The details in this manuscript describe the plan for this trial as per the currently active trial protocol Version 4.0 01/03/2023. Recruitment of the first participant into the trial took place on 15/02/2023. Recruitment will continue until July 2026. The DMC will review safety data for the trial when the first 100–150 participants have been randomised (expected around September 2024) to inform the request for amendments to the protocol from the regulators (eg, modifications to the clinical monitoring schedule to make it less onerous for participants and study doctors). If deemed appropriate based on the initial trial safety data, this could facilitate faster recruitment and higher retention in the trial.

## Data Availability

Data sharing not applicable as no datasets generated and/or analysed for this study.
