# Supplementary material for: Ashwagandha (Withania somnifera (L.) Dunal) for promoting recovery in long covid: protocol for a randomised placebo-controlled clinical trial (APRIL Trial)
Source: BMJ Open. 2025 Apr 25;15(4):e094526. doi: 10.1136/bmjopen-2024-094526 (PMC12035422; doi:10.1136/bmjopen-2024-094526)
Supplement: Supplementary file 1 [file bmjopen-2024-094526.reviewer_comments.pdf]

PEER REVIEW HISTORY

BMJ Open publishes all reviews undertaken for accepted manuscripts. Reviewers are asked to complete a checklist review form and are provided with free text boxes to elaborate on their assessment. These free text comments are reproduced below.

ARTICLE DETAILS

Title (Provisional)

Ashwagandha (Withania somnifera (L.) Dunal) for promoting recovery in Long COVID: Protocol for a randomised placebo-controlled clinical trial (APRIL Trial)

Authors

Mallinson, Poppy Alice Carson; Joshi, Manisha; Mathpathi, Mahesh; Perkins, Alexander; Clayton, Tim; Shah, Anoop SV; Mathur, Rohini; Birk, Nick; Dhillon, Arandeep; Lieber, Judith; Beg, Sidra S; Hopkins, Lily; Khan, Archie; Allaham, Shereen; Kam, Vanessa TW; Sutaria, Shailen; R, Galib; Rajagopala, S; Bhamra, Amarjeet; Pillai, Geetha Krishnan G; Khunti, Kamlesh; Nesari, Tanuja; Kinra, Sanjay

---

VERSION 1 - REVIEW

**Reviewer**	**1**
**Name**	**Ling, CQ**
**Affiliation**	**School of Traditional Chinese Medicine, Naval Medical University**
**Date**	**15-Jan-2025**
**COI**	**None**

---

1. Eligibility criteria {10} section: The current inclusion criteria mention "Have been diagnosed with Long COVID as per the NICE Guidelines (NG188)," but it does not specify whether the severity of the patient's symptoms should be considered. It is recommended to add consideration of the severity of Long COVID symptoms in the inclusion criteria.

In the Introduction, the authors stated that “There are currently no known effective treatment options for Long COVID.” At the same time, it should be emphasized the various efforts that are currently underway. It is recommended to introduce the current efforts and research on the late-stage treatment of COVID and Long COVID to deepen the significance of the article. Most recent references, such as PMID: 36717302, 38937157, etc., could be cited.

3. Intervention description {11a} section: Although it mentions the dosage form, packaging, etc., of Ashwagandha and placebo, the specific environment and auxiliary conditions for participants to take the medication are not detailed enough. It is suggested to supplement

the details of the environment and auxiliary conditions when participants take Ashwagandha root extract tablets.

4. Criteria for discontinuing or modifying allocated interventions {11b} section: The current standards for discontinuing or modifying intervention measures mainly focus on liver function abnormalities and serious adverse events, but there is less mention of monitoring other physiological indicators that may affect the patient's overall health. It is recommended to add regular monitoring of other important physiological functions such as renal function (such as blood creatinine, urea nitrogen, etc.) and electrocardiogram (ECG) for participants, and clarify the corresponding abnormal value handling standards to ensure the safety of participants more comprehensively.

5. Data management {19} and Confidentiality {27} sections: Although it mentions the principles of data storage and confidentiality, the specific allocation of data access rights, data sharing rules at different stages of the study, and the execution details of confidentiality measures are not described in detail. It is suggested to clearly state which research team members can access the original data under what circumstances, and the specific process and restrictions of data sharing at different stages such as data analysis and paper writing. At the same time, for confidentiality measures, the encryption and anonymization operations of participant personal information during data transmission and storage should be detailed.

6. Statistical methods for primary and secondary outcomes {20a} section: Although it mentions that intention-to-treat analysis and other statistical methods will be used, it does not explain how to deal with potential confounding factors and how to conduct multivariate analysis to more accurately assess the effect of Ashwagandha. It is suggested to supplement the explanation of which potential confounding factors (such as age, gender, underlying diseases, etc.) will be considered in the statistical analysis, and what kind of multivariate analysis method (such as multivariate linear regression, logistic regression, etc.) will be used to adjust the impact of these confounding factors, so as to more accurately assess the independent effect of Ashwagandha on the functional status and symptom burden of Long COVID patients.

**Reviewer**	**2**
**Name**	**Lan, Chou-Chin**
**Affiliation**	**Division of Pulmonary Medicine, Taipei Tzu Chi Hospital**
**Date**	**27-Jan-2025**
**COI**	**None**

The APRIL trial aims to evaluate the efficacy and safety of Ashwagandha for improving functional status and alleviating symptoms in adults with Long COVID. The trial plans to

recruit 2,500 participants and will primarily assess outcomes using the Post-COVID Functional Status Scale (PCFSS) after three months, employing baseline-adjusted ordinal logistic regression analysis. Monthly online surveys will be conducted to collect patient-reported outcomes and monitor safety. The study has received ethics and regulatory approval, and results will be disseminated through peer-reviewed publications and conference presentations. If found effective, the results could influence treatment guidelines and improve health outcomes for patients with Long COVID.

The APRIL trial aims to evaluate the efficacy and safety of Ashwagandha (Withania somnifera) for promoting recovery among adults with Long COVID. The study plans to recruit 2,500 participants who will be randomized to receive either Ashwagandha root extract or a matching placebo for three months. Primary outcomes will be assessed using the Post-COVID Functional Status Scale (PCFSS) at the three-month mark through baseline-adjusted ordinal logistic regression analysis. Additionally, monthly online surveys will gather patient-reported outcomes and conduct safety monitoring, including liver function tests. The trial has received necessary ethical and regulatory approvals, and results will be disseminated through peer-reviewed publications and conference presentations. If found effective, the findings could significantly influence treatment guidelines and improve health outcomes for those affected by Long COVID.

However, although the abstract outlines the study design, it lacks a deeper description of the expected outcomes and statistical data, which may lead readers to question the actual significance of the results.

The introduction highlights that as many as 45% of individuals worldwide may suffer from long-term symptoms of long COVID, underscoring the necessity and significance of research. It introduces common symptoms among patients with long COVID, such as fatigue, muscle weakness, and respiratory issues, providing readers with comprehensive background information. The potential severe impacts of long COVID on healthcare systems and the economy are mentioned, further emphasizing the importance of the research. The urgency of finding new treatment options is stressed, given the current lack of effective therapies for long COVID.

However, the description lacks specific data support: While it mentions a high proportion of long COVID patients experiencing symptoms, more detailed data is absent, which may reduce its persuasiveness. Although some references are provided, more supporting studies might be needed relative to the issues discussed to enhance the authority of the introduction.

Method:

The study adopts a randomized double-blind controlled design, which effectively reduces bias and enhances the credibility of the results. This design ensures that participants are unaware of their group assignments, helping to control the influence of psychological factors on the outcomes. The study explicitly outlines inclusion and exclusion criteria, ensuring

participant homogeneity and enhancing the external validity of the trial results. Monthly online surveys and clinical monitoring provide continuous data collection, aiding in the assessment of participants' health status and treatment effectiveness. However, the study includes only clinically diagnosed long COVID patients, which may limit the generalizability of the findings, especially when extrapolating to self-diagnosed cases. While many potential confounding factors, such as the use of other herbal medicines, are excluded, verifying the complete elimination of such confounding variables remains a challenge to overcome.

The study's outcomes offer several advantages and drawbacks. On the positive side, the comprehensive nature of the results is a strength, as it includes various dimensions such as quality of life, self-reported fatigue, pain, sleep, anxiety, and depression, all of which are common symptoms in long COVID patients. This allows for a well-rounded assessment of their health status. Additionally, the study is patient-centered, with all primary and secondary outcomes based on patient-reported data, which ensures that their subjective experiences are given significant weight, providing a more accurate reflection of their true feelings. The use of validated measurement tools, such as the PROMIS scale, further enhances the validity and credibility of the findings. However, there are some limitations. The reliance on self-reported data introduces the potential for bias, as participants' subjective feelings may influence the results, limiting the reliability of the data. Furthermore, the study's dependence on online or mailed surveys for data collection could pose a barrier for patients who have difficulty accessing the internet or participating in the survey, which may affect the generalizability of the results.

The study has several advantages regarding its timeline and sample size. The timeline is well-organized, with the trial planned to be completed within 91 days, allowing participants to receive results in a relatively short time. This is particularly important for long COVID patients, as they are often eager to see improvements in their symptoms. The sample size is also adequate, with plans to recruit 2,500 participants. This ensures the trial's statistical power, which boosts confidence in detecting treatment effects. Moreover, such a sample size enables a comprehensive understanding of symptom improvement and provides meaningful data. Despite the reasonable timeline, large-scale recruitment may face challenges, especially when targeting patients with specific conditions like long COVID, making it difficult to recruit enough participants within the planned timeframe. There is also the potential for participant attrition. Although the trial anticipates limited loss to follow-up, there is still a risk of participants dropping out, which could impact the final results and conclusions. Additionally, if the sample is predominantly from specific regions or populations, it may limit the generalizability and applicability of the results, restricting the broader impact of the findings.

Data Collection and Management Methods: The trial's data collection and management methods have several advantages and disadvantages. On the positive side, secure data storage ensures that participant information from screening, registration, and follow-up forms is stored on secure servers, protecting sensitive data in accordance with regulations,

which enhances trust in the trial process and data integrity. Data monitoring is another advantage, as the database includes mechanisms to flag abnormal data values, allowing the trial team to contact participants for clarification, helping to maintain data quality and resolve potential inconsistencies in a timely manner. However, there are drawbacks. The reliance on electronic systems for data input and management could present challenges, such as technical issues or data loss, and system failures may interfere with data collection and analysis. Participant engagement may also be limited; while follow-up calls are planned, non-respondents or those not participating in online platforms could lead to incomplete data, affecting the overall quality of the dataset. Lastly, potential bias in the data collection process, especially through self-reported questionnaires, may arise if participants misunderstand questions or are influenced by their current emotional or health state, which could impact the reliability of self-reported outcomes.

Discussion Section: The discussion section has several advantages and drawbacks. On the positive side, the study's necessity is clearly addressed, as it focuses on the needs of long COVID patients, highlighting the current lack of effective treatments and proposing Ashwagandha as a potential therapy, helping to fill a medical gap. The emphasis on patient involvement is another strength, as the study design takes into account the opinions and needs of long COVID patients, refining the research methods through interaction with patients and healthcare providers, showing a strong focus on patient needs. Additionally, the rigorous monitoring and design, including multi-center recruitment and comprehensive clinical monitoring procedures, help ensure data reliability and safety, enhancing the credibility of the study results. However, there are some drawbacks. The diagnostic limitation of including only diagnosed long COVID patients may restrict the applicability of the results to all long COVID patients, especially undiagnosed cases. Participant restrictions may also be an issue, as some patients with severe activity limitations may be unable to attend clinical sites for necessary monitoring, leading to insufficient samples and potential bias in the results. Finally, the generalizability of the results is uncertain, as the study's design and sample sources may limit the ability to apply the findings to all types of long COVID patients, particularly those in the most severe symptom stages.

The manuscript should include a Limitations of the Study section to address the inherent constraints of the research. One limitation is the diagnostic focus, as the study includes only diagnosed long COVID patients, which may limit the applicability of the findings to undiagnosed cases or those with less clearly defined symptoms. Another limitation is participant accessibility; some long COVID patients with severe activity restrictions may not be able to attend clinical sites for necessary monitoring, potentially leading to a sample that does not fully represent the broader patient population. Additionally, the generalizability of the results may be compromised if the sample population is not fully representative of all long COVID patients, particularly those in more severe stages of the disease. The reliance on self-reported data is another limitation, as it could introduce bias based on participants' subjective perceptions, affecting the objectivity of the findings. Finally, the dependence on

electronic systems for data collection and management introduces the risk of technical issues, such as data loss or system failures, which could undermine the data's integrity. Addressing these limitations would provide transparency and a more balanced interpretation of the study's findings.

VERSION 1 - AUTHOR RESPONSE

Reviewer: 1. Dr. CQ Ling, School of Traditional Chinese Medicine, Naval Medical University

1. Eligibility criteria {10} section: The current inclusion criteria mention "Have been diagnosed with Long COVID as per the NICE Guidelines (NG188)," but it does not specify whether the severity of the patient's symptoms should be considered. It is recommended to add consideration of the severity of Long COVID symptoms in the inclusion criteria.

- Severity is already part of the inclusion criteria, because to be eligible people must report that Long COVID has “reduced their ability to carry out day-to-day activities compared with the time before they had COVID-19” (p7). Thus people with mild, non-activity-limiting cases of Long COVID would not be eligible.

2. In the Introduction, the authors stated that “There are currently no known effective treatment options for Long COVID.” At the same time, it should be emphasized the various efforts that are currently underway. It is recommended to introduce the current efforts and research on the late-stage treatment of COVID and Long COVID to deepen the significance of the article. Most recent references, such as PMID: 36717302, 38937157, etc., could be cited.

- Thank you, we agree that there is relevant recent evidence in this area and we could engage more with this literature. We have added the following lines in the introduction (p5): “While a few trials have indicated potential benefits of rehabilitation or cognitive behavioural therapy (8), the development of pharmaceutical targets for Long COVID has met limited success(9). The latest systematic review identified four randomised trials of drug interventions, which tested a disparate range of treatments (monoclonal antibodies, antidepressants, muramyl peptides, and a calf blood extract) none of which provided clear evidence of benefit for Long COVID patients(8).”

3. Intervention description {11a} section: Although it mentions the dosage form, packaging, etc., of Ashwagandha and placebo, the specific environment and auxiliary conditions for participants to take the medication are not detailed enough. It is suggested to supplement the details of the environment and auxiliary conditions when participants take Ashwagandha root extract tablets.

- Thank you, the following details were already mentioned in this paragraph: “Participants will be requested to take two tablets each morning and two tablets each evening. Tablets should be swallowed whole and if available a glass of warm water can be used to wash them down (following Ayurvedic recommendations).” (p10). We have now added the following details for extra clarification (p10): “If they miss a dose of medication, they will be asked not to catch this up another time, and to note the missed dose in the medication diary. Written instructions for how to take the medication will be provided to participants.”

4. Criteria for discontinuing or modifying allocated interventions {11b} section: The current standards for discontinuing or modifying intervention measures mainly focus on liver function

abnormalities and serious adverse events, but there is less mention of monitoring other physiological indicators that may affect the patient's overall health. It is recommended to add regular monitoring of other important physiological functions such as renal function (such as blood creatinine, urea nitrogen, etc.) and electrocardiogram (ECG) for participants, and clarify the corresponding abnormal value handling standards to ensure the safety of participants more comprehensively.

- This trial already has regulatory approval and has started recruitment. Monitoring of these clinical parameters was not deemed proportionate to the risk profile and known effects of this study IMP by the study team or by the Medicines and Healthcare Regulatory Agency (UK MHRA), who conducted thorough reviews of this product's safety profile to inform their decision. Investigators based at clinical sites are able to conduct any additional medical monitoring that they deem indicated (including ECG and other blood tests, which is described at more length in the full approved Trial Protocol V4 section 4.3 Clinical Monitoring (p23), which we will attach as an Appendix).

5. Data management {19} and Confidentiality {27} sections: Although it mentions the principles of data storage and confidentiality, the specific allocation of data access rights, data sharing rules at different stages of the study, and the execution details of confidentiality measures are not described in detail. It is suggested to clearly state which research team members can access the original data under what circumstances, and the specific process and restrictions of data sharing at different stages such as data analysis and paper writing. At the same time, for confidentiality measures, the encryption and anonymization operations of participant personal information during data transmission and storage should be detailed.

- Thank you, we agree these points are vital for trial integrity; they are detailed at length in our Data Management Plan and other trial-specific SOPs and other documentation. To briefly expand on these in the manuscript, we have added the following text (p17): "These data systems will be access-controlled with access only provided to necessary study staff such as those entering and monitoring the data, and trial staff based at study sites will only able to view information pertaining to participants from their site.... Once the trial data entry is completed and all data queries have been satisfactorily resolved, the database will be locked down by the trial manager by revoking site staffs' access and downloading the final version of the database as an un-editable file stored on the Sponsor's secure servers."; and (p18): "These data extracts will be de-identified because no participant identifers (e.g. name, date of birth) will be included, and participants' Trial IDs will be replaced with new random IDs. They will be extracted from the main locked-down study database by the Trial Manager and shared with the statistical analyst via shared folders within the Sponsor's secure servers. Handling of all data will be in accordance with standards set out in the UK National Health System's Data Security and Protection Toolkit."

6. Statistical methods for primary and secondary outcomes {20a} section: Although it mentions that intention-to-treat analysis and other statistical methods will be used, it does not explain how to deal with potential confounding factors and how to conduct multivariate analysis to more accurately assess the effect of Ashwagandha. It is suggested to supplement the explanation of which potential confounding factors (such as age, gender, underlying diseases, etc.) will be considered in the statistical analysis, and what kind of multivariate analysis method (such as multivariate linear regression, logistic regression, etc.) will be used to adjust the impact of these confounding factors, so as to more accurately assess the independent effect of Ashwagandha on the functional status and symptom burden of Long COVID patients.

- Thank you, this detail is forthcoming in our statistical analysis plan which will be published before the database is locked down and unblinding occurs. We did not plan to conduct adjusted analysis as our primary analysis as this is a large simple trial and potential confounding factors should be balanced by randomisation; however we agree that if baseline covariate imbalances were noted, it might be prudent to include secondary analyses with adjustment for such covariates; we have added a line to specify this (p19): "Further secondary analyses will include multivariate models that adjust for baseline covariates: age, sex, presence of cardiometabolic co-morbidity, and other baseline factors which exhibit imbalance between trial arms at baseline (should there be any)."

Reviewer: 2. Dr. Chou-Chin Lan, Division of Pulmonary Medicine, Taipei Tzu Chi Hospital

The APRIL trial aims to evaluate the efficacy and safety of Ashwagandha for improving functional status and alleviating symptoms in adults with Long COVID. The trial plans to recruit 2,500 participants and will primarily assess outcomes using the Post-COVID Functional Status Scale (PCFSS) after three months, employing baseline-adjusted ordinal logistic regression analysis. Monthly online surveys will be conducted to collect patient-reported outcomes and monitor safety. The study has received ethics and regulatory approval, and results will be disseminated through peer-reviewed publications and conference presentations. If found effective, the results could influence treatment guidelines and improve health outcomes for patients with Long COVID.

The APRIL trial aims to evaluate the efficacy and safety of Ashwagandha (Withania somnifera) for promoting recovery among adults with Long COVID. The study plans to recruit 2,500 participants who will be randomized to receive either Ashwagandha root extract or a matching placebo for three months. Primary outcomes will be assessed using the Post-COVID Functional Status Scale (PCFSS) at the three-month mark through baseline-adjusted ordinal logistic regression analysis. Additionally, monthly online surveys will gather patient-reported outcomes and conduct safety monitoring, including liver function tests. The trial has received necessary ethical and regulatory approvals, and results will be disseminated through peer-reviewed publications and conference presentations. If found effective, the findings could significantly influence treatment guidelines and improve health outcomes for those affected by Long COVID. However, although the abstract outlines the study design, it lacks a deeper description of the expected outcomes and statistical data, which may lead readers to question the actual significance of the results.

The introduction highlights that as many as 45% of individuals worldwide may suffer from long-term symptoms of long COVID, underscoring the necessity and significance of research. It introduces common symptoms among patients with long COVID, such as fatigue, muscle weakness, and respiratory issues, providing readers with comprehensive background information. The potential severe impacts of long COVID on healthcare systems and the economy are mentioned, further emphasizing the importance of the research. The urgency of finding new treatment options is stressed, given the current lack of effective therapies for long COVID.

However, the description lacks specific data support: While it mentions a high proportion of long COVID patients experiencing symptoms, more detailed data is absent, which may reduce its persuasiveness. Although some references are provided, more supporting studies might be needed relative to the issues discussed to enhance the authority of the introduction.

- These specifics are given and referenced in the introduction (p4). We have expanded on the evidence on other treatments of Long COVID for which evidence has progressed since we first submitted this manuscript (p5).

Method:

The study adopts a randomized double-blind controlled design, which effectively reduces bias and enhances the credibility of the results. This design ensures that participants are unaware of their group assignments, helping to control the influence of psychological factors on the outcomes. The study explicitly outlines inclusion and exclusion criteria, ensuring participant homogeneity and enhancing the external validity of the trial results. Monthly online surveys and clinical monitoring provide continuous data collection, aiding in the assessment of participants' health status and treatment effectiveness. However, the study includes only clinically diagnosed long COVID patients, which may limit the generalizability of the findings, especially when extrapolating to self-diagnosed cases. While many potential confounding factors, such as the use of other herbal medicines, are excluded, verifying the complete elimination of such confounding variables remains a challenge to overcome.

The study's outcomes offer several advantages and drawbacks. On the positive side, the comprehensive nature of the results is a strength, as it includes various dimensions such as quality of life, self-reported fatigue, pain, sleep, anxiety, and depression, all of which are common symptoms in long COVID patients. This allows for a well-rounded assessment of their health status. Additionally, the study is patient-centered, with all primary and secondary outcomes based on patient-reported data, which ensures that their subjective experiences are given significant weight, providing a more accurate reflection of their true feelings. The use of validated measurement tools, such as the PROMIS scale, further enhances the validity and credibility of the findings. However, there are some limitations. The reliance on self-reported data introduces the potential for bias, as participants' subjective feelings may influence the results, limiting the reliability of the data. Furthermore, the study's dependence on online or mailed surveys for data collection could pose a barrier for patients who have difficulty accessing the internet or participating in the survey, which may affect the generalizability of the results.

The study has several advantages regarding its timeline and sample size. The timeline is well-organized, with the trial planned to be completed within 91 days, allowing participants to receive results in a relatively short time. This is particularly important for long COVID patients, as they are often eager to see improvements in their symptoms. The sample size is also adequate, with plans to recruit 2,500 participants. This ensures the trial's statistical power, which boosts confidence in detecting treatment effects. Moreover, such a sample size enables a comprehensive understanding of symptom improvement and provides meaningful data. Despite the reasonable timeline, large-scale recruitment may face challenges, especially when targeting patients with specific conditions like long COVID, making it difficult to recruit enough participants within the planned timeframe. There is also the potential for participant attrition. Although the trial anticipates limited loss to follow-up, there is still a risk of participants dropping out, which could impact the final results and conclusions. Additionally, if the sample is predominantly from specific regions or populations, it may limit the generalizability and applicability of the results, restricting the broader impact of the findings.

Data Collection and Management Methods: The trial's data collection and management methods have several advantages and disadvantages. On the positive side, secure data storage ensures that participant information from screening, registration, and follow-up forms is stored on secure servers, protecting sensitive data in accordance with regulations, which enhances trust in the trial process and data integrity. Data monitoring is another advantage, as the

database includes mechanisms to flag abnormal data values, allowing the trial team to contact participants for clarification, helping to maintain data quality and resolve potential inconsistencies in a timely manner. However, there are drawbacks. The reliance on electronic systems for data input and management could present challenges, such as technical issues or data loss, and system failures may interfere with data collection and analysis. Participant engagement may also be limited; while follow-up calls are planned, non-respondents or those not participating in online platforms could lead to incomplete data, affecting the overall quality of the dataset. Lastly, potential bias in the data collection process, especially through self-reported questionnaires, may arise if participants misunderstand questions or are influenced by their current emotional or health state, which could impact the reliability of self-reported outcomes.

Discussion Section: The discussion section has several advantages and drawbacks. On the positive side, the study's necessity is clearly addressed, as it focuses on the needs of long COVID patients, highlighting the current lack of effective treatments and proposing Ashwagandha as a potential therapy, helping to fill a medical gap. The emphasis on patient involvement is another strength, as the study design takes into account the opinions and needs of long COVID patients, refining the research methods through interaction with patients and healthcare providers, showing a strong focus on patient needs. Additionally, the rigorous monitoring and design, including multi-center recruitment and comprehensive clinical monitoring procedures, help ensure data reliability and safety, enhancing the credibility of the study results. However, there are some drawbacks. The diagnostic limitation of including only diagnosed long COVID patients may restrict the applicability of the results to all long COVID patients, especially undiagnosed cases. Participant restrictions may also be an issue, as some patients with severe activity limitations may be unable to attend clinical sites for necessary monitoring, leading to insufficient samples and potential bias in the results. Finally, the generalizability of the results is uncertain, as the study's design and sample sources may limit the ability to apply the findings to all types of long COVID patients, particularly those in the most severe symptom stages.

The manuscript should include a Limitations of the Study section to address the inherent constraints of the research. One limitation is the diagnostic focus, as the study includes only diagnosed long COVID patients, which may limit the applicability of the findings to undiagnosed cases or those with less clearly defined symptoms. Another limitation is participant accessibility; some long COVID patients with severe activity restrictions may not be able to attend clinical sites for necessary monitoring, potentially leading to a sample that does not fully represent the broader patient population. Additionally, the generalizability of the results may be compromised if the sample population is not fully representative of all long COVID patients, particularly those in more severe stages of the disease. The reliance on self-reported data is another limitation, as it could introduce bias based on participants' subjective perceptions, affecting the objectivity of the findings. Finally, the dependence on electronic systems for data collection and management introduces the risk of technical issues, such as data loss or system failures, which could undermine the data's integrity. Addressing these limitations would provide transparency and a more balanced interpretation of the study's findings.

- There is already a strengths and limitations paragraph which covers these points. Patient-reported measures are the target outcome of the study; we do not perceive there to be an 'objectivity' concern here. Bias that may arise due to patients' knowledge of their treatment affecting their reporting is not a concern due to the placebo-controlled design. Electronic systems are an accepted standard for clinical trial data management.

Our systems are rigorously backed-up and change-controlled and compliant with all regulatory and UK NHS requirements.
